# Supplementary material for: Socioeconomic Inequalities Impact the Ability of Pregnant Women and Women of Childbearing Age to Consume Nutrients Needed for Neurodevelopment: An Analysis of NHANES 2007–2018
Source: Nutrients. 2022 Sep 16;14(18):3823. doi: 10.3390/nu14183823 (PMC9500720; doi:10.3390/nu14183823)
Supplement: Supplementary file 1 [file nutrients-14-03823-s001.zip › nutrients-1889234-supplementary.pdf]

nutrients

MDPI

Socioeconomic Inequalities Impact the Ability of Pregnant Women and Women of Childbearing Age to Consume Nutrients Needed for Neurodevelopment; An Analysis of NHANES 2007-2018

Supplementary Material

Supplementary Table S1. Total usual intakes of nutrients important for neurodevelopment (foods + dietary supplements) among non*pregnant nonlactating* US women aged 20-44 y in the NHANES Survey by SNAP participation.

	Household Recipient of SNAP in Past 12 Months			
	Yes, N=1,617		No, N=4,031	
**Nutrient**	**Mean (SE)**	**% <EAR or [AI]**	**Mean (SE)**	**% <EAR or [AI]**
Vitamin A, RAE/d	688 (32.3)	47.0 (2.80)	975 (40.1) *	28.3 (2.05) *
Vitamin B6, mg/d	3.12 (0.24)	6.96 (1.50)	4.46 (0.25) *	3.41 (0.82) *
Vitamin B12, ug/d	31.4 (5.58)	--	43.5 (5.90) *	--
Vitamin C, mg/d	100 (4.63)	40.1 (2.18)	151 (8.26) *	24.4 (1.48) *
Vitamin D, mg/d	7.46 (0.36)	83.8 (1.04)	13.3 (1.17) *	71.1 (0.97) *
Vitamin E, mg/d	8.11 (0.45)	93.3 (1.10)	13.7 (1.97) *	84.5 (1.75) *
Vitamin K, ug/d	101 (3.19)	[47.6 (2.18)]	131 (4.15) *	[26.0 (2.70)] *
Zinc, mg/d	11.4 (0.20)	40.2 (2.25)	13.4 (0.23) *	30.7 (1.74) *
Iron, mg/d	15.8 (0.43)	6.45 (1.19)	17.8 (0.31) *	4.25 (0.88) *
Choline, mg/d	280 (4.94)	[96.7 (0.88)]	296 (3.65) *	[94.8 (1.04)]
Folate, ug DFE/d	567 (12.8)	12.8 (1.77)	702 (14.0) *	7.49 (1.29) *
Calcium, mg/d	929 (14.8)	37.5 (1.96)	1037 (14.2) *	26.9 (1.57) *
Magnesium, mg/d	253 (3.73)	58.1 (1.91)	300 (3.81) *	37.5 (1.60) *
EPA + DHA, mg/d	46.9 (4.29)	95.0 (0.72)	65.8 (4.81) *	93.6 (0.59)

* indicates $p<0.05$ relative to those in households that received SNAP. % below for EPA + DHA represents those below recommendations for intake from the Dietary Guidelines for Americans.

Supplementary Table S2. Data on total usual intakes of nutrients important for neurodevelopment (foods alone) among *nonpregnant nonlactating* US women aged 20-44 in the National Health and Nutrition Examination Survey by PIR.

	Low, N=2,763		Medium, N=1,209		High, N=1,336	
**Nutrient**	**Mean (SE)**	**% <EAR or [AI]**	**Mean (SE)**	**% <EAR or [AI]**	**Mean (SE)**	**% <EAR or [AI]**
Vitamin A, RAE/d	555 (14.1) [a]	45.1 (2.60) [d]	638 (18.3) [b]	31.3 (3.10) [e]	680 (19.3) [c]	25.4 (3.03) [f]
Vitamin B6, mg/d	1.84 (0.03)	11.0 (1.88)	1.98 (0.05)	7.59 (1.79)	2.03 (0.04)	6.72 (1.54)
Vitamin B12, ug/d	4.55 (0.11)	--	4.73 (0.12)	--	4.78 (0.12)	--
Vitamin C, mg/d	75.6 (2.26) [a]	41.2 (2.34) [e]	81.6 (2.89) [b]	35.5 (2.84) [f]	87.8 (2.46) [c]	30.2 (2.26) [g]
Vitamin D, mg/d	4.13 (0.10) [a]	98.3 (0.40) [d]	4.33 (0.14) [b]	97.9 (0.46) [e]	4.40 (0.14) [b]	97.7 (0.48) [e]
Vitamin E, mg/d	7.57 (0.16) [a]	93.4 (1.13) [d]	8.60 (0.23) [b]	87.2 (2.06) [e]	9.28 (0.22) [c]	81.8 (2.27) [f]
Vitamin K, ug/d	105 (3.53) [a]	[43.1 (3.22)] [d]	121 (4.12) [b]	[30.6 (3.25)] [e]	138 (5.72) [c]	[20.3 (3.55)] [f]
Zinc, mg/d	9.78 (0.13) [a]	46.1 (2.07) [d]	10.2 (0.20) [a]	38.6 (3.21) [e]	10.5 (0.16) [ab]	34.3 (2.69) [e]
Iron, mg/d	12.9 (0.16) [a]	3.97 (0.77) [d]	13.3 (0.24) [ab]	3.02 (0.69) [e]	13.8 (0.21) [b]	1.94 (0.52) [f]
Choline, mg/d	279 (3.79) [a]	[97.1 (0.70)] [d]	298 (6.14) [b]	[94.9 (1.32)] [e]	300 (5.23) [b]	[94.7 (1.34)] [e]
Folate, ug DFE/d	474 (8.56) [a]	13.5 (1.91) [d]	496 (11.7) [b]	10.6 (1.68) [e]	517 (11.6) [c]	8.22 (1.81) [f]
Calcium, mg/d	875 (12.3) [a]	39.2 (1.57) [d]	930 (15.9) [b]	32.2 (2.50) [e]	954 (15.5) [c]	28.8 (2.48) [f]
Magnesium, mg/d	253 (3.86) [a]	56.2 (2.18) [d]	275 (4.37) [b]	44.4 (2.39) [e]	296 (4.82) [c]	34.0 (2.35) [f]

EPA + DHA, mg/d	47.4 (3.30) ^{a}	95.8 (0.52) ^{b}	49.5 (4.25) ^{ab}	96.1 (0.65) ^{b}	57.9 (4.20) ^{b}	94.9 (0.77) ^{b}

Rows with different subscripts differ from each other, p<0.05, low 0-1.85, medium >1.85-3.50, high >3.5. % below for EPA + DHA represents those below recommendations for intake from the Dietary Guidelines for Americans.

Supplementary Table S3. Data on total usual intakes of nutrients important for neurodevelopment (foods + dietary supplements) among *nonpregnant nonlactating* US women aged 20-44 in the National Health and Nutrition Examination Survey by PIR.

	**Low, N=2,763**		**Medium, N=1,209**		**High, N=1,336**	
**Nutrient**	**Mean (SE)**	**% <EAR or [AI]**	**Mean (SE)**	**% <EAR or [AI]**	**Mean (SE)**	**% <EAR or [AI]**
Vitamin A, RAE/d	803 (46.7) ^{a}	41.7 (2.42)^{d}	937 (64.0)^{b}	28.4 (2.81)^{e}	1027 (59.2)^{c}	22.0 (2.69)^{f}
Vitamin B6, mg/d	3.19 (0.22) ^{a}	5.66 (1.27)^{c}	4.41 (0.40)^{a}	3.25 (0.96)^{c}	5.29 (0.44)^{ab}	2.46 (0.74)^{cd}
Vitamin B12, ug/d	48.0 (12.0) ^{a}	--	38.9 (7.05)^{b}	--	37.8 (5.81)^{b}	--
Vitamin C, mg/d	112 (5.33) ^{a}	34.0 (1.96)^{c}	156 (19.1)^{a}	26.2 (2.18)^{c}	165 (14.9)^{b}	21.1 (1.59)^{cd}
Vitamin D, mg/d	8.70 (0.75) ^{a}	80.3 (1.11)^{d}	13.4 (3.21)^{b}	73.0 (1.50)^{e}	15.6 (1.39)^{c}	66.7 (1.54)^{f}
Vitamin E, mg/d	9.28 (0.72) ^{a}	92.1 (1.20)^{d}	14.1 (3.15)^{b}	85.8 (2.07)^{e}	15.9 (4.00)^{c}	79.9 (2.43)^{f}
Vitamin K, ug/d	108 (3.58) ^{a}	[41.0 (3.09)]^{d}	125 (4.32)^{b}	[28.3 (3.03)]^{e}	146 (5.80)^{c}	[18.0 (3.11)]^{f}
Zinc, mg/d	11.8 (0.21) ^{b}	39.3 (1.87)^{d}	13.1 (0.32)^{b}	30.3 (2.60)^{e}	14.2 (0.33)^{c}	25.6 (2.14)^{f}
Iron, mg/d	16.3 (0.34) ^{a}	6.18 (1.12)^{d}	17.4 (0.48)^{b}	4.96 (1.13)^{e}	18.7 (0.45)^{c}	3.31 (0.92)^{f}
Choline, mg/d	280 (3.79) ^{a}	[97.0 (0.71)]^{c}	303 (6.47)^{b}	[94.3 (1.34)]^{d}	303 (6.13)^{b}	[94.3 (1.42)]^{d}
Folate, ug DFE/d	594 (13.0) ^{a}	11.5 (1.60)^{d}	699 (23.5)^{b}	8.15 (1.28)^{e}	752 (19.2)^{c}	5.88 (1.34)^{f}
Calcium, mg/d	948 (14.0) ^{a}	35.1 (1.78)^{d}	1038 (19.1)^{b}	26.2 (2.13)^{e}	1091 (20.0)^{c}	22.1 (2.08)^{f}
Magnesium, mg/d	266 (4.51) ^{a}	52.4 (2.12)^{d}	291 (5.05)^{b}	40.2 (2.30)^{e}	324 (5.78)^{c}	29.2 (2.09)^{f}
EPA + DHA, mg/d	50.2 (4.64) ^{a}	94.8 (0.68)^{c}	54.4 (5.56)^{a}	95.0 (0.83)^{c}	74.1 (7.80)^{b}	92.8 (0.97)^{c}

Rows with different subscripts differ from each other, p<0.05, -- indicates suppression due to relative standard errors >30%, low 0-1.85, medium >1.85-3.50, high >3.5. % below for EPA + DHA represents those below recommendations for intake from the Dietary Guidelines for Americans.

Supplementary Table S4. Data on total usual intakes of nutrients important for neurodevelopment (foods alone) among *nonpregnant nonlactating* US women aged 20-44 in the National Health and Nutrition Examination Survey by household food security .

	**Very low, N=539**		**Low, N=945**		**Marginal, N=805**		**Full, N=3,374**	
**Nutrient**	**Mean (SE)**	**% <EAR or [AI]**	**Mean (SE)**	**% <EAR or [AI]**	**Mean (SE)**	**% <EAR or [AI]**	**Mean (SE)**	**% <EAR or [AI]**
Vitamin A, RAE/d	541 (16.0)^{a}	47.7 (2.93)^{d}	543 (16.7)^{a}	47.8 (3.02)^{d}	578 (22.8)^{b}	41.2 (4.05)^{e}	643 (14.6)^{c}	31.0 (2.39)^{f}
Vitamin B6, mg/d	1.84 (0.04)^{a}	12.1 (2.00)^{c}	1.84 (0.04) ^{a}	11.7 (1.89) ^{c}	1.85 (0.05) ^{a}	10.8 (2.13)^{d}	1.98 (0.03)^{b}	7.69 (1.49)^{e}
Vitamin B12, ug/d	4.46 (0.11)^{a}	--	4.45 (0.12) ^{a}	--	4.65 (0.70) ^{a}	--	4.72 (0.10)^{b}	--
Vitamin C, mg/d	70.6 (2.46)^{a}	46.2 (2.72) ^{d}	70.9 (2.60) ^{a}	46.2 (2.83) ^{d}	76.6 (3.31) ^{b}	40.1 (3.37) ^{e}	85.3 (1.94) ^{c}	32.1 (1.86)^{f}
Vitamin D, mg/d	4.00 (0.12)^{a}	98.7 (0.38)^{d}	4.01 (0.12)^{a}	98.6 (0.38)^{d}	4.20 (0.17)^{b}	98.3 (0.51)^{d}	4.36 (0.11)^{c}	97.8 (0.41)^{d}
Vitamin E, mg/d	7.44 (0.18)^{a}	93.9 (1.28)^{d}	7.47 (0.19)^{a}	93.4 (1.31)^{d}	8.14 (0.30)^{b}	89.9 (2.07)^{e}	8.69 (0.16)^{c}	86.2 (1.71)^{f}
Vitamin K, ug/d	101 (3.35)^{a}	[47.0 (3.01)]^{d}	101 (3.51)^{a}	[47.0 (3.06)]^{d}	116 (5.86)^{b}	[34.4 (4.60)]^{e}	125 (3.99)^{c}	[28.3 (2.96)]^{f}
Zinc, mg/d	9.76 (0.18)^{a}	46.2 (3.13)^{b}	9.76 (0.18)^{a}	46.6 (3.12)^{b}	9.99 (0.24)^{a}	42.3 (4.06)^{b}	10.3 (0.12)^{a}	38.3 (2.01)^{b}
Iron, mg/d	12.8 (0.22)^{a}	3.48 (0.81)^{c}	12.8 (0.23)^{a}	3.63 (0.80)^{c}	13.0 (0.33)^{a}	3.83 (0.88)^{d}	13.5 (0.14)^{b}	2.55 (0.54)^{e}
Choline, mg/d	280 (5.74)^{a}	[96.9 (0.89)]^{c}	280 (5.86)^{a}	[96.7 (0.92)]^{c}	288 (7.16)^{a}	[96.0 (1.03)]^{c}	294 (3.94)^{b}	[95.1 (1.07)]^{c}
Folate, ug DFE/d	466 (9.94)^{a}	14.5 (2.18)^{c}	466 (10.2)^{a}	14.4 (2.18)^{c}	478 (14.7)^{a}	12.4 (2.26)^{cd}	504 (8.28)^{ab}	9.21 (1.64)^{d}
Calcium, mg/d	858 (15.2)^{a}	43.1 (2.37)^{c}	861 (15.7)^{a}	42.9 (2.41)^{c}	896 (11.9)^{b}	37.2 (3.72)^{c}	929 (10.8)^{b}	32.5 (1.85)^{d}
Magnesium, mg/d	252 (4.76)^{a}	56.8 (2.67)^{c}	253 (4.99)^{a}	56.8 (2.86)^{c}	260 (5.94)^{a}	52.5 (3.30)^{c}	280 (3.15)^{b}	41.9 (1.58)^{d}
EPA + DHA, mg/d	47.7 (10.4)^{b}	95.6 (1.38)^{b}	53.2 (2.65)^{a}	95.8 (0.81)^{b}	47.8 (5.97)^{b}	95.7 (0.88)^{b}	49.1 (4.95)^{b}	95.3 (0.46)^{b}

-- indicates suppression due to relative standard errors >30%, rows with different subscripts differ from each other, p<0.05. % below for EPA + DHA represents those below recommendations for intake from the Dietary Guidelines for Americans.

Supplementary Table S5. Data on total usual intakes of nutrients important for neurodevelopment (foods + dietary supplements) among *nonpregnant nonlactating* US women aged 20-44 in the National Health and Nutrition Examination Survey by household food security .

Nutrient	Very low, N=539		Low, N=945		Marginal, N=805		Full, N=3,374	
	Mean (SE)	% <EAR or [AI]	Mean (SE)	% <EAR or [AI]	Mean (SE)	% <EAR or [AI]	Mean (SE)	% <EAR or [AI]
Vitamin A, RAE/d	685 (42.7)^{a}	48.3 (3.66)^{d}	748 (48.7)^{ab}	32.3 (3.25)^{e}	813 (70.5)^{b}	38.2 (3.79)^{e}	989 (42.3)^{c}	27.6 (2.11)^{f}
Vitamin B6, mg/d	2.73 (0.21)^{a}	7.52 (1.77)^{c}	3.30 (0.36)^{a}	5.19 (1.23)^{d}	2.99 (0.21)^{a}	5.62 (1.41)^{d}	4.71 (0.29)^{b}	3.35 (0.85)^{e}
Vitamin B12, ug/d	24.9 (5.67)^{a}	3.93 (1.57)	48.7 (10.6)^{b}	--	32.2 (8.64)^{ab}	--	42.6 (6.67)^{b}	--
Vitamin C, mg/d	109 (13.7)^{a}	44.0 (3.41)^{c}	130 (24.9)^{ab}	34.6 (2.81)^{d}	124 (10.1)^{ab}	32.4 (2.91)^{d}	148 (7.76)^{b}	23.6 (1.45)^{e}
Vitamin D, mg/d	10.8 (3.53)^{a}	82.9 (2.15)^{d}	8.10 (0.55)^{b}	79.9 (1.69)^{d}	9.40 (0.79)^{ab}	79.7 (2.19)^{d}	13.4 (1.29)^{c}	70.6 (1.08)^{e}
Vitamin E, mg/d	8.43 (1.08)^{a}	94.2 (1.37)^{d}	8.53 (0.53)^{a}	91.7 (1.59)^{d}	10.2 (1.54)^{b}	89.4 (2.09)^{de}	14.1 (2.27)^{c}	83.9 (1.79)^{f}
Vitamin K, ug/d	96.0 (3.88)^{a}	[51.6 (3.55)]^{d}	109 (4.24)^{a}	[41.1 (3.36)]^{d}	120 (6.07)^{b}	[32.5 (4.48)]^{e}	131 (4.11)^{c}	[25.8 (2.68)]^{f}
Zinc, mg/d	11.2 (0.38)^{a}	44.9 (4.28)^{c}	12.1 (0.37)^{a}	37.3 (3.17)^{c}	12.2 (0.37)^{a}	35.4 (3.34)^{d}	13.4 (0.23)^{b}	30.0 (1.67)^{e}
Iron, mg/d	15.9 (0.86)^{a}	8.36 (1.87)^{c}	16.8 (0.67) ^{a}	5.21 (1.27)^{d}	16.7 (0.65)^{a}	5.40 (1.26)^{d}	17.8 (0.29)^{b}	4.04 (0.85)^{d}
Choline, mg/d	271 (7.32)^{a}	[97.7 (0.78)]^{c}	288 (7.95)^{a}	[95.6 (1.34)]^{c}	289 (7.21)^{b}	[95.8 (1.06)]^{c}	297 (3.92)^{b}	[94.8 (1.08)]^{d}
Folate, ug DFE/d	562 (27.8)^{a}	15.8 (2.71)^{d}	605 (18.9)^{b}	10.4 (1.86)^{e}	609 (22.0)^{b}	10.1 (1.85)^{e}	709 (14.5)^{c}	6.97 (1.27)^{f}
Calcium, mg/d	919 (27.4)^{a}	39.7 (3.33)^{c}	953 (21.9)^{ab}	35.2 (2.50)^{c}	978 (25.5)^{b}	32.2 (3.29)^{d}	1042 (14.3)^{b}	26.3 (1.62)^{e}
Magnesium, mg/d	254 (6.50)^{a}	58.1 (3.21)^{d}	271 (6.82)^{b}	50.8 (3.17)^{e}	276 (7.66)^{a}	48.6 (3.34)^{e}	301 (3.65)^{bc}	37.2 (1.49)^{f}
EPA + DHA, mg/d	48.6 (10.3)^{b}	95.2 (1.39)^{c}	54.0 (9.25)^{ab}	95.1 (0.84)^{c}	46.4 (5.04)^{b}	96.0 (0.79)^{c}	64.2 (4.31)^{a}	93.4 (0.60)^{c}

Rows with different subscripts differ from each other, p<0.05, -- indicates suppression due to relative standard errors >30%. % below for EPA + DHA represents those below recommendations for intake from the Dietary Guidelines for Americans.

Supplementary Table S6. Total usual intakes of nutrients important for neurodevelopment (foods + dietary supplements) among *pregnant nonlactating* US women aged 20-44 y in the NHANES Survey by WIC participation.

	Household recipient of WIC in past 12 months			
	Yes, N=124		No, N=191	
Nutrient	Mean (SE)	% <EAR or [AI]	Mean (SE)	% <EAR or [AI]
Vitamin A, RAE/d	1484 (315)	27.1 (7.36)	1755 (292)	18.9 (5.10)
Vitamin B6, mg/d	4.20 (0.59)	–	–	–
Vitamin B12, ug/d	9.24 (0.78)	–	–	–
Vitamin C, mg/d	149 (13.8)	22.8 (4.74)	181 (12.9)*	11.4 (2.59)*
Vitamin D, mg/d	10.7 (1.27)	56.9 (6.33)	14.1 (1.08)*	41.8 (4.53)*
Vitamin E, mg/d	9.41 (1.37)	90.7 (3.92)	10.5 (0.65)	79.9 (6.28)
Vitamin K, ug/d	103 (12.0)	[47.8 (10.3)]	148 (13.7)*	–
Zinc, mg/d	18.9 (1.40)	19.0 (6.06)	21.3 (1.02)	11.9 (3.05)*
Iron, mg/d	28.8 (2.37)	54.1 (6.57)	35.6 (3.05)*	38.4 (4.37)*
Choline, mg/d	298 (16.8)	[97.2 (2.50)]	311 (13.9)	[96.1 (2.97)]
Folate, ug DFE/d	1191 (107)	23.2 (6.99)	1477 (79.1)*	14.4 (2.84)*
Calcium, mg/d	1234 (90.2)	15.5 (5.04)	1337 (62.3)	–
Magnesium, mg/d	286 (13.7)	55.0 (7.22)	325 (13.0)*	38.5 (5.82)*
EPA + DHA, mg/d	52.9 (15.0)	95.0 (2.39)	83.8 (21.1)*	94.0 (1.91)*

-- indicates suppression due to relative standard errors >30%, *indicates p<0.05 relative to those in households that received WIC. % below for EPA + DHA represents those below recommendations for intake from the Dietary Guidelines for Americans.

Supplementary Table S7. Total usual intakes of nutrients important for neurodevelopment (foods + dietary supplements) among *pregnant nonlactating* US women aged 20-44 y in the NHANES Survey by SNAP participation.

	Household recipient of SNAP in past 12 months

Nutrient	Yes, N=112 Mean (SE)	Yes, N=112 % <EAR or [AI]	No, N=204 Mean (SE)	No, N=204 % <EAR or [AI]
Vitamin A, RAE/d				
Vitamin B6, mg/d	3.09 (0.33)	–	11.9 (4.46)	–
Vitamin B12, ug/d	7.70 (0.74)	–	–	–
Vitamin C, mg/d	125 (10.0)	29.6 (5.51)	186 (12.9)*	10.2 (2.22)*
Vitamin D, mg/d	8.78 (0.98)	67.0 (5.78)	14.4 (1.13)*	39.5 (4.19)*
Vitamin E, mg/d	7.34 (0.64)	96.3 (2.95)	11.1 (0.76)*	79.1 (6.14)*
Vitamin K, ug/d	87.0 (9.99)	[60.6 (9.80)]	149 (13.6)*	–
Zinc, mg/d	17.1 (1.43)	–	22.0 (0.99)*	–
Iron, mg/d	24.8 (2.05)	65.5 (5.68)	36.8 (2.83)*	34.9 (4.27)*
Choline, mg/d	285 (18.9)	[98.4 (2.32)]	315 (13.2)*	96.0 (2.98)*
Folate, ug DFE/d	948 (90.9)	33.7 (8.79)	1556 (74.2)*	–
Calcium, mg/d	1169 (73.1)	–	1372 (67.3)*	–
Magnesium, mg/d	275 (15.9)	65.7 (7.10)	327 (10.8)*	35.3 (5.38)*
EPA + DHA, mg/d	47.3 (11.3)	96.4 (1.78)	87.6 (21.9)*	93.3 (2.04)*

-- indicates suppression due to relative standard errors >30%, *indicates $p<0.05$ relative to those in households who received SNAP, % below for EPA + DHA represents those below recommendations for intake from the Dietary Guidelines for Americans.

Supplementary Table S8. Data on total usual intakes of nutrients important for neurodevelopment (foods alone) among *pregnant nonlactating* US women aged 20-44 in the National Health and Nutrition Examination Survey by PIR.

Nutrient	Low, N=162 Mean (SE)	Low, N=162 % <EAR or [AI]	Medium, N=60 Mean (SE)	Medium, N=60 % <EAR or [AI]	High, N=70 Mean (SE)	High, N=70 % <EAR or [AI]
Vitamin A, RAE/d	639 (44.5)[a]	38.4 (9.28)[c]	740 (73.4)[b]	--	765 (73.0)[b]	--
Vitamin B6, mg/d	1.92 (0.10)[a]	31.2 (7.99)[b]	1.99 (0.14)[a]	26.0 (8.38)[b]	2.17 (0.17)[a]	16.8 (5.71)[c]
Vitamin B12, ug/d	4.99 (0.38)[a]	--	4.74 (0.45)[a]	--	5.23 (0.47)[a]	--
Vitamin C, mg/d	101 (7.40)[a]	33.0 (6.24)[b]	114 (16.6)[a]	--	114 (12.0)[a]	23.7 (6.66)[b]
Vitamin D, mg/d	5.49 (0.51)[a]	93.2 (3.25)[b]	5.21 (0.75)[a]	94.5 (3.98)[b]	5.16 (0.54)[a]	94.7 (3.16)[b]
Vitamin E, mg/d	7.15 (0.48)[a]	96.6 (2.43)[c]	9.64 (0.69)[b]	81.3 (8.35)[d]	10.1 (0.77)[b]	75.6 (9.54)[d]
Vitamin K, ug/d	93.7 (9.62)[a]	[54.3 (9.92)]	144 (14.2)[b]	--	156 (18.0)[b]	--
Zinc, mg/d	10.6 (0.59)[a]	--	11.7 (0.91)[b]	--	11.9 (0.65)[b]	--
Iron, mg/d	15.2 (0.92)[a]	96.6 (3.27)[b]	16.4 (1.35)[a]	94.7 (5.61)[b]	16.6 (1.02)[a]	93.0 (6.24)[b]
Choline, mg/d	291 (16.2)[a]	[96.9 (2.49)][b]	315 (19.6)[c]	[95.2 (3.75)][b]	315 (21.2)[c]	[94.5 (2.83)][b]
Folate, ug DFE/d	547 (39.4)[c]	47.3 (10.8)[c]	635 (65.5)[c]	27.2 (14.1)[d]	617 (42.2)[a]	31.8 (10.6)[d]
Calcium, mg/d	1098 (56.5)[a]	--	1164 (97.1)[a]	--	1111 (73.4)[a]	--
Magnesium, mg/d	275 (11.8)[a]	61.2 (6.22)[c]	319 (16.6)[b]	37.3 (8.66)[d]	313 (16.3)[b]	42.7 (8.54)[d]
EPA + DHA, mg/d	47.9 (7.47)[a]	97.3 (0.87)[bc]	49.4 (12.9)[a]	94.5 (2.97)[b]	44.1 (13.2)[a]	99.2 (0.84)[bc]

-- indicates suppression due to relative standard errors >30%, rows with different subscripts differ from each other, $p<0.05$, low 0-1.85, medium >1.85-3.50, high >3.5. % below for EPA + DHA represents those below recommendations for intake from the Dietary Guidelines for Americans.

Supplementary Table S9. Data on total usual intakes of nutrients important for neurodevelopment (foods + dietary supplements) among *pregnant nonlactating* US women aged 20-44 in the National Health and Nutrition Examination Survey by PIR.

Nutrient	Low, N=162 Mean (SE)	Low, N=162 % <EAR or [AI]	Medium, N=60 Mean (SE)	Medium, N=60 % <EAR or [AI]	High, N=70 Mean (SE)	High, N=70 % <EAR or [AI]
Vitamin A, RAE/d	1059 (111)[a]	34.4 (7.52)	1168 (144)[ab]	–	2211 (511)[b]	–
Vitamin B6, mg/d	5.56 (1.45)[a]	–	–	–	7.52 (1.24)[b]	–
Vitamin B12, ug/d	–	–	15.3 (3.81)	–	–	–
Vitamin C, mg/d	162 (15.9)[a]	23.6 (4.63)	175 (19.8)[a]	–	179 (16.7)[a]	–

Vitamin D, mg/d	10.3 (1.07)^{a}	61.5 (5.05)^{c}	18.7 (2.68)^{b}	29.4 (7.22)^{d}	12.6 (1.28)^{a}	43.8 (6.66)^{e}
Vitamin E, mg/d	8.30 (1.12)^{a}	95.4 (2.54)^{c}	10.3 (0.78)^{b}	78.8 (8.20)^{c}	12.0 (1.03)^{b}	71.2 (8.76)^{d}
Vitamin K, ug/d	96.1 (9.81)^{a}	[52.7 (9.66)]	158 (15.6)^{b}	–	160 (18.2)^{b}	–
Zinc, mg/d	96.1 (9.81)^{a}	52.7 (9.66)	158 (15.6)^{b}	–	160 (18.2)^{b}	–
Iron, mg/d	26.2 (2.31)^{a}	64.9 (5.03)^{c}	36.0 (3.12)^{b}	26.1 (6.33)^{f}	38.8 (5.47)^{b}	32.4 (6.65)^{e}
Choline, mg/d	291 (16.1)^{a}	[96.8 (2.50)]^{b}	316 (19.5)^{a}	[96.8 (2.50)^{b}	317 (21.2)^{a}	[94.4 (3.97)]^{b}
Folate, ug DFE/d	1038 (82.9)^{a}	30.7 (7.24)^{c}	1567 (117)^{b}	–	1647 (121)^{b}	10.4 (3.46)^{d}
Calcium, mg/d	1200 (58.6)^{a}	–	1389 (133)^{a}	–	1312 (85.3)^{a}	–
Magnesium, mg/d	282 (12.5)^{a}	58.3 (6.21)^{c}	338 (18.5)^{b}	29.8 (8.46)^{d}	326 (16.7)^{ab}	38.1 (7.83)^{cd}
EPA + DHA, mg/d	57.3 (10.4)^{a}	95.2 (1.72)^{c}	50.6 (10.9)^{a}	95.5 (2.29)^{c}	104 (35.3)^{b}	93.6 (2.86)^{c}

Rows with different subscripts differ from each other, $p<0.05$, -- indicates suppression due to relative standard errors >30%. % below for EPA + DHA represents those below recommendations for intake from the Dietary Guidelines for Americans.

Supplementary Table S10. Data on total usual intakes of nutrients important for neurodevelopment (foods alone) among *pregnant nonlactating* US women aged 20-44 in the National Health and Nutrition Examination Survey by household food security .

	Very low to low, N=99		Marginal, N=41		Full, N=176	
**Nutrient**	**Mean (SE)**	**% <EAR or [AI]**	**Mean (SE)**	**% <EAR or [AI]**	**Mean (SE)**	**% <EAR or [AI]**
Vitamin A, RAE/d	648 (64.8)^{a}	--	567 (63.4)^{b}	50.7 (12.5)	796 (57.1)^{a}	--
Vitamin B6, mg/d	1.87 (0.12)^{ab}	--	1.67 (0.16)^{a}	47.8 (14.4)^{c}	2.18 (0.12)^{b}	12.5 (5.98)^{d}
Vitamin B12, ug/d	4.93 (0.56)^{ab}	--	4.10 (0.54)^{b}	--	5.56 (0.38)^{a}	--
Vitamin C, mg/d	81.3 (9.24)^{a}	48.4 (9.05)^{d}	99.1 (13.8)^{b}	34.9 (9.97)^{d}	120 (9.61)^{c}	20.4 (4.62)^{e}
Vitamin D, mg/d	4.95 (0.55)^{ab}	96.1 (2.38)^{cd}	4.40 (0.72)^{a}	97.6 (2.36)^{c}	5.61 (0.43)^{b}	93.5 (3.21)^{d}
Vitamin E, mg/d	8.26 (0.69)^{ab}	90.9 (4.49)^{cd}	7.97 (0.89)^{b}	92.6 (5.96)^{d}	9.34 (0.57)^{a}	83.1 (6.64)^{c}
Vitamin K, ug/d	104 (13.0)^{a}	--	115 (23.1)^{ab}	--	139 (13.5)^{b}	--
Zinc, mg/d	10.8 (0.74)^{ab}	--	9.10 (0.94)^{a}	60.3 (22.5)	12.2 (0.48)^{b}	--
Iron, mg/d	14.8 (1.11)^{c}	99.1 (1.77)^{de}	12.2 (1.40)^{b}	100 (0.37)^{e}	17.4 (0.75)^{a}	94.2 (6.19)^{d}
Choline, mg/d	283 (19.0)^{ab}	[98.4 (2.05)]^{cd}	274 (32.1)^{a}	[98.6 (2.33)]^{d}	321 (14.8)^{b}	[95.7 (3.37)]^{c}
Folate, ug DFE/d	564 (54.9)^{a}	42.2 (18.6)^{d}	464 (58.2)^{b}	68.4 (18.2)^{e}	636 (32.0)^{c}	25.3 (11.5)^{f}
Calcium, mg/d	1074 (74.8)^{ab}	--	987 (112)^{b}	--	1169 (51.4)^{a}	51.4 (11.0)
Magnesium, mg/d	271 (10.3)^{ab}	63.6 (6.05)^{cd}	266 (18.9)^{b}	68.2 (11.0)^{d}	317 (12.4)^{a}	38.4 (7.43)^{c}
EPA + DHA, mg/d	30.9 (8.45)^{a}	98.9 (0.79)^{b}	--	91.8 (5.86)^{b}	47.7 (13.3)^{a}	98.2 (0.78)^{b}

-- indicates suppression due to relative standard errors >30%, rows with different subscripts differ from each other, $p<0.05$. % below for EPA + DHA represents those below recommendations for intake from the Dietary Guidelines for Americans.

Supplementary Table S11. Data on total usual intakes of nutrients important for neurodevelopment (foods + dietary supplements) among *pregnant nonlactating* US women aged 20-44 in the National Health and Nutrition Examination Survey by household food security .

	Very low to low, N=70		Marginal, N=60		Full, N=162	
**Nutrient**	**Mean (SE)**	**% <EAR or [AI]**	**Mean (SE)**	**% <EAR or [AI]**	**Mean (SE)**	**% <EAR or [AI]**
Vitamin A, RAE/d	1382 (353)^{ab}	31.5 (10.7)^{c}	1004 (261)^{b}	46.1 (11.5)^{d}	1916 (308)^{b}	12.1 (5.26)^{e}
Vitamin B6, mg/d	3.58 (0.51)^{a}	–	5.57 (1.07)^{b}	–	12.8 (5.31)^{c}	–
Vitamin B12, ug/d	10.1 (1.77)^{a}	–	9.42 (1.12)^{a}	–	–	–
Vitamin C, mg/d	136 (16.5)^{a}	30.1 (4.95)^{d}	149 (16.1)^{b}	15.3 (6.07)^{e}	188 (14.0)^{c}	8.76 (2.53)^{e}
Vitamin D, mg/d	9.31 (0.99)^{a}	62.8 (6.99)^{c}	12.4 (1.99)^{a}	44.1 (10.7)^{c}	14.6 (1.29)^{b}	40.4 (4.86)^{d}
Vitamin E, mg/d	8.30 (0.69)^{ab}	90.6 (4.47)^{d}	11.3 (3.59)^{a}	90.3 (6.34)^{c}	10.7 (0.72)^{a}	79.2 (6.30)^{c}
Vitamin K, ug/d	105 (13.2)^{b}	[45.6 (10.5)]^{d}	129 (25.8)^{a}	[34.3 (14.7)]^{c}	145 (13.6)^{a}	[21.9 (8.08)]^{c}
Zinc, mg/d	18.1 (1.74)^{a}	–	16.9 (2.12)^{a}	26.5 (8.15)	22.5 (1.08)^{b}	–
Iron, mg/d	27.5 (3.02)^{a}	60.4 (8.41)^{c}	24.9 (3.14)^{a}	52.6 (10.5)^{d}	37.7 (3.37)^{b}	34.9 (4.66)^{e}
Choline, mg/d	283 (19.1)^{a}	[98.4 (2.05)]^{b}	275 (32.0)^{a}	[98.6 (2.39)]^{b}	322 (14.9)^{a}	[95.5 (3.44)]^{b}

Folate, ug DFE/d	982 (172)[a]	–	1189 (119)[a]	26.2 (8.14)	1565 (90.3)[b]	–
Calcium, mg/d	1275 (124)[ab]	–	1096 (135)[a]	–	1377 (63.0)[b]	–
Magnesium, mg/d	287 (14.9)[a]	59.4 (6.26)[c]	276 (20.9)[a]	64.7 (11.4)[c]	332 (13.3)[b]	32.5 (6.91)[d]
EPA +DHA, mg/d	87.4 (22.0)[a]	97.1 (1.85)[c]	73.5 (31.3)[ab]	93.9 (4.44)[cd]	40.4 (11.4)[b]	93.3 (2.02)[d]

Rows with different subscripts differ from each other, p<0.05, -- indicates suppression due to relative standard errors >30%. % below for EPA + DHA represents those below recommendations for intake from the Dietary Guidelines for Americans.
